# The Question Mark Question: Wound Healing after Hemicraniectomy

**DOI:** 10.1055/a-2561-7951

**Published:** 2025-04-09

**Authors:** Grace R. Fassina, Emily E. Nguyen, David Cho, Drake J. Williams, Alexander R. Evans, Panayiotis E. Pelargos, Christopher S. Graffeo

**Affiliations:** 1 Department of Neurosurgery, University of Oklahoma, Oklahoma City, Oklahoma, United States; 2 Department of Neurosurgery, Washington University in St. Louis, St. Louis, Missouri, United States

**Keywords:** reverse question mark, retroauricular, wound complications, decompressive hemicraniectomy

## Abstract

**Introduction:**

The reverse question mark (RQM) incision is the conventional scalp flap technique incorporated during decompressive hemicraniectomy (DHC) operations. Recently, the retroauricular (RA) incision emerged as a possible alternative. We sought to assess the contemporary literature regarding postoperative outcomes following RQM or RA for DHC and subsequent cranioplasty.

**Methods:**

MEDLINE and Embase databases were queried using the Preferred Reporting Items for Systematic Reviews and Meta-Analyses (PRISMA) guidelines. Studies reporting outcomes after primary DHC and secondary cranioplasty for both RQM and RA were included in a meta-analysis of proportions performed using random-effects modeling.

**Results:**

Five manuscripts representing a total of 511 patients met the inclusion criteria. No difference was observed in the rate of primary surgical site infection (SSI) after RQM as compared with RA (OR = 1.64, 95% CI = 0.92–2.92). Similarly, based on those four studies reporting SSI data after cranioplasty, no difference was detected in secondary SSI rates between RQM and RA (OR = 1.93, 95% CI = 0.79–4.72). Patients who underwent primary RQM had increased odds of undergoing cranioplasty compared with patients who had received a primary RA (OR = 1.57, 95% CI = 1.03–2.39).

**Conclusion:**

This novel systematic review and meta-analysis reported postoperative outcomes after DHC using either the RQM or RA incision technique. No significant difference was noted in SSI after either the primary decompression or the secondary cranioplasty. These findings support the use of either technique in routine practice, as guided by surgeon preference or other clinical considerations, such as superficial temporal artery preservation for an unrelated future indication.

## Introduction

Decompressive hemicraniectomy (DHC) is a commonly performed neurological surgery involving the removal of a bone flap to manage high intracranial pressure. Frequent pathologies leading to a DHC include traumatic brain injury with or without intracranial hemorrhage and malignant cerebral edema secondary to large-volume infarction, although numerous other indications have been reported. Most surviving patients subsequently undergo cranioplasty to repair the skull defect, typically at a delay of 3 to 6 months following the initial operation. Complications following DHC and cranioplasty are fairly common, with an overall estimated event rate of 10% for routine complications, including postoperative surgical site infection (SSI), cerebrospinal fluid (CSF) leak, or postoperative hemorrhage. 1

**Fig. 1:**
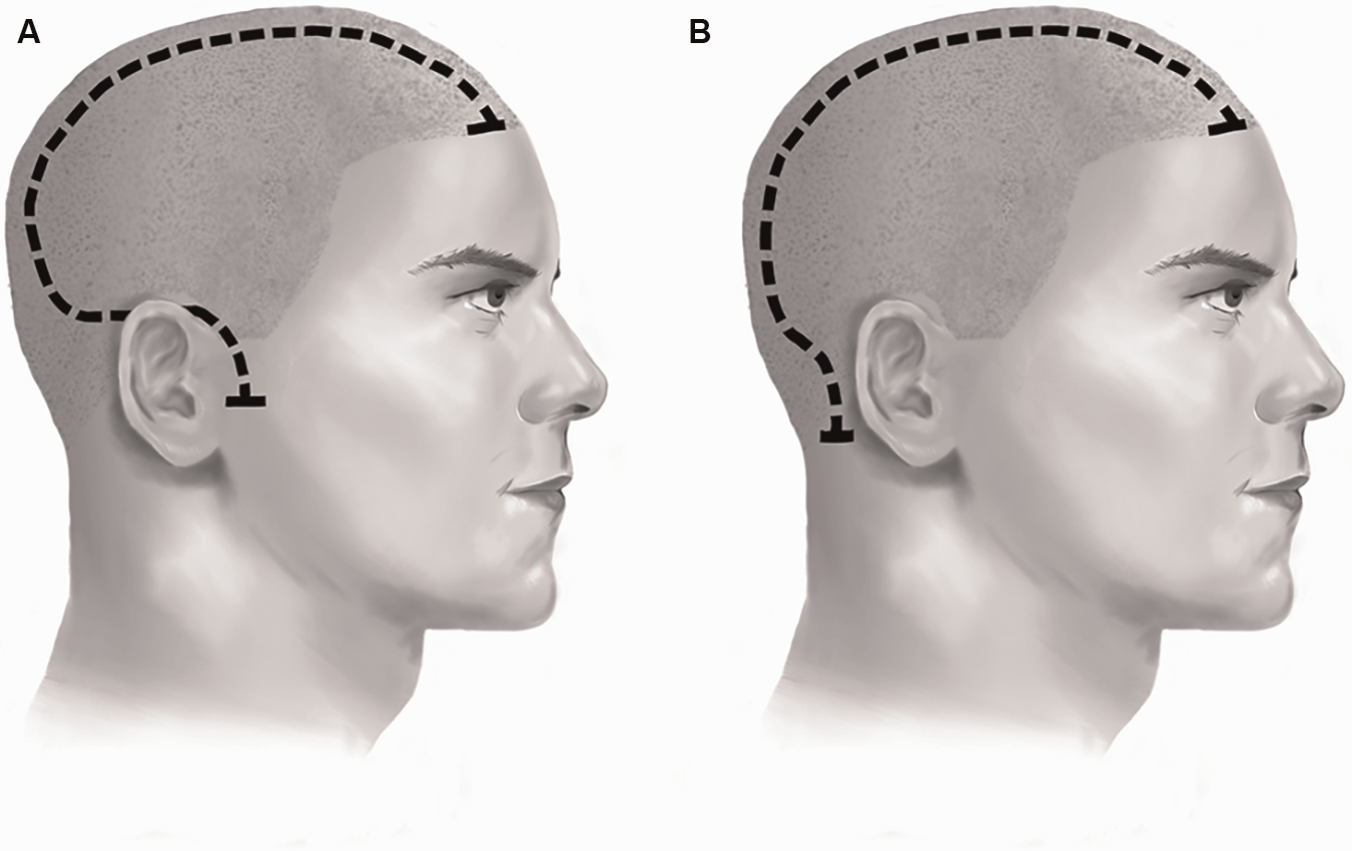
Illustration comparing right-sided techniques for ( **A** ) the standard reverse question mark (RQM) incision, as compared with ( **B** ) the retroauricular (RA) incision for decompressive hemicraniectomy.

Most neurosurgeons employ the conventional reverse question mark (RQM) incision for DHC operations. 2 3 The RQM is planned starting just above the zygomatic root and 1 cm anterior to the tragus, curving superiorly and posteriorly around the helix of the pinna before coursing posteriorly toward the occiput and completing a wide U-shaped curve that places the superior limb of the incision parallel to and 1 to 2 cm lateral to the midline until the hairline is reached ( Fig. 1a ). Although this is considered a reliable and versatile technique, the relatively high incidence of wound complications and the associated risk of superficial temporal artery (STA) injury have prompted various investigators to propose and study alternative DHC incisions. 4 5 These incisions include the L.G. Kempe (T-bar) incision and the retroauricular (RA) incision.

The RA incision, also referred to as the “posterior altered question mark,” shares the superior and posterior limbs with the RQM, but rather than coursing anteriorly and following the curvature of the pinna, the RA takes a retroauricular pathway to terminate posteriorly to the base of the mastoid process ( Fig. 1b ). This technical modification spares the STA, theoretically providing a flap with a more robust blood supply and lower risk of wound complications, including SSI. 3 6 Preliminary observational evidence has been equivocal, although Veldeman et al., 7 in 2020, reported a significantly reduced SSI rate after RA as compared with RQM for DHC operations. With these considerations in mind, we sought to perform a systematic review and meta-analysis of the literature comparing RQM and RA incisions for DHC.

## Methods

### Search Strategy and Data Collection

The current study was conducted in compliance with the Preferred Reporting Items for Systematic Reviews and Meta-Analyses (PRISMA) guidelines. We queried MEDLINE and Embase from inception through November 2023 using the MeSH terms (“Decompressive Craniectomy” or “Craniotomy”) or “decompressive hemicraniectomy” or “hemicraniectomy” or “craniectomy” or “craniotomy” AND “question mark” or “retroauricular” or “retro-auricular.” All studies reporting and comparing outcomes after RQM and RA in adult patients (≥18 years) undergoing DHC were included. Case studies, conference abstracts, non-English language publications, and review articles were excluded. The primary search yielded 62 candidate citations, of which 16 underwent full-text review; 5 manuscripts met all study criteria and were included. The primary study outcome was SSI after primary DHC; the secondary outcome was SSI after subsequent cranioplasty.

### Statistical Analysis

Statistical pooling of the included study results was performed using a meta-analysis of proportions with random-effects modeling. 8 Pooled and weighted effect sizes were calculated with 95% confidence intervals (CIs) and reported with conventional forest plots. Heterogeneity was assessed by calculating *I* ^2^ . The risk of bias was assessed using funnel plots and Egger's regression test.

## Results

### Overview of the Study Sample

Five studies were included in this review and meta-analysis, comprising 511 patients, of whom 305 underwent RQM and 206 underwent RA ( Table 1 ). 9 10 The mean age was 51.0 years (range 46.9–56.38), and the percentage of female patients was 34.6%. Among the five included studies that reported a direct comparison between RQM and RA outcomes, four also reported the proportion of patients proceeding to eventual cranioplasty, time-to-cranioplasty, and secondary SSI after cranioplasty ( Table 2 ).

**Table 1:** Characteristics of studies included

Study	Study design	Size (number)	Number of Female	Mean age (years)
Hoffman et al. 2023 6	Cohort study	110	33	46.9
Früh et al., 2023 9	Cohort study	69	41	52
Dowlati et al., 2021 3	Cohort study	106	42	50.25
Veldeman et al., 2020 7	Cohort study	166	61	49.5
Nerntengian et al., 2022 10	Cohort study	60	n/a	56.38

**Table 2:** Clinical features and outcomes

Study	Number	Age (mean)	Number of females	SSI post-DHC	Time to cranioplasty (days)	Total number of cranioplasty	SSI postcranioplasty
Retroauricular incision							
Hoffman et al., 2023 6	27	46	4	1	135	21	0
Früh et al., 2023 9	27	54	9	5	133	16	2
Dowlati et al., 2021 3	43	49	16	2	127	21	1
Veldeman et al., 2020 7	79	47	32	6	90	21	5
Nerntengian et al., 2022 10	30	56	N/A	8	N/A	N/A	N/A
Reverse question mark incision							
Hoffman et al., 2023 6	83	47	25	4	135	60	4
Früh et al., 2023 9	42	50	32	12	113	34	2
Dowlati et al., 2021 3	63	52	26	5	111	40	3
Veldeman et al., 2020 7	87	52	29	9	90	31	16
Nerntengian et al., 2022 10	30	57	n/a	12	N/A	N/A	N/A

Abbreviations: DHC, decompressive hemicraniectomy; SSI, surgical site infection.

### Primary or Secondary Surgical Site Infection after Reverse Question Mark versus Retroauricular

**Fig. 2:**
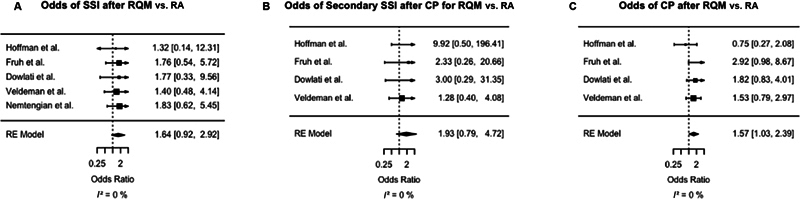
Forest plots depicting the individual and weighted odds ratios ( **A** ) for primary surgical site infection (SSI) after RQM versus RA incisions for DHC; ( **B** ) for undergoing cranioplasty after RQM versus RA incisions for DHC; and ( **C** ) for secondary SSI after cranioplasty with RQM versus RA incision. DHC, decompressive hemicraniectomy; RA, retroauricular; RQM, reverse question mark.

No difference was observed in the rate of primary surgical site infection (SSI) after RQM as compared with RA (OR = 1.64, 95% CI = 0.92–2.92; Table 3 and Fig. 2A ). Similarly, based on those four studies reporting SSI data after cranioplasty, no difference was detected in secondary SSI rates between RQM and RA (OR = 1.93, 95% CI = 0.79–4.72; Fig. 2B ). Patients who underwent primary RQM had an increased odds of undergoing cranioplasty, as compared with patients who had received a primary RA (OR = 1.57, 95% CI = 1.03–2.39; Fig. 2C ). Heterogeneity was low across all pooled analyses ( *I* ^2^ = 0%).

**Table 3:** Reported outcomes

Study	Odds of SSI after RQM versus RA OR (95% CI)	Odds of CP after RQM versus RA OR (95% CI)	Odds of SSI after CP in RQM versus RA OR (95% CI)
Hoffman et al., 2023 6	1.32 (0.14, 12.31)	0.75 (0.72, 2.08)	9.92 (0.50, 196.41)
Früh et al., 2023 9	1.76 (0.54, 5.72)	2.92 (0.98, 8.67)	2.33 (0.26, 20.66)
Dowlati et al., 2021 3	1.77 (0.33, 9.56)	1.82 (0.83, 4.01)	3.00 (0.29, 31.35)
Veldeman et al., 2020 7	1.40 (0.48, 4.14)	1.53 (0.79, 2.97)	1.28 (0.40, 4.08)
Nerntengian et al., 2022 10	1.83 (0.62, 5.45)	N/A	N/A

Abbreviations: DHC, decompressive hemicraniectomy; RA, retroauricular; RQM, reverse question mark; SSI, surgical site infection.

### Risk-of-Bias Assessment

**Fig. 3:**
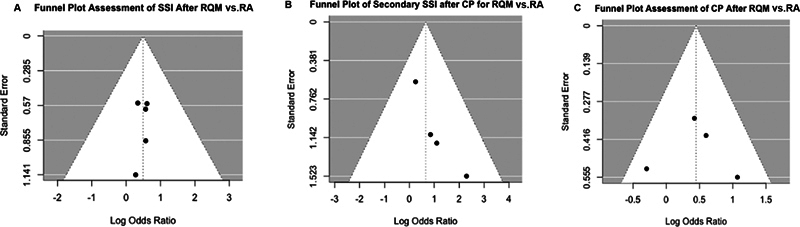
Funnel plots graphically assessing the risk of publication bias in the comparisons for ( **A** ) primary surgical site infection (SSI) after RQM versus RA incisions for DHC; ( **B** ) cranioplasty after RQM versus RA incisions for DHC; and ( **C** ) secondary SSI after cranioplasty with RQM versus RA incision. DHC, decompressive hemicraniectomy; RA, retroauricular; RQM, reverse question mark.

Funnel plots were symmetrical, indicating a low risk of publication bias, which was confirmed by the non-significant result on Egger's and Begg's tests ( Fig. 3A–C ).

## Discussion

We report a novel meta-analysis comparing SSI and cranioplasty rates after RQM versus RA techniques for DHC and secondary cranioplasty. In pooled analysis, we observed broadly comparable SSI results after both primary and secondary operations independent of the technique used. A significantly larger fraction of RQM patients went on to cranioplasty as compared with RA; however, this finding is likely unrelated to the operative technique used during DHC. Overall, these results are broadly supportive of both techniques as safe, effective options for neurosurgeons performing DHC operations on adult patients.

### Findings of Studies Analyzed

Among the five manuscripts meeting the criteria for inclusion in the current study, four failed to identify any significant difference between the RQM and RA techniques with regard to postoperative healing or wound complications. Only Veldeman et al. 7 demonstrated a significant difference between the groups, which was specific to the secondary SSI rates after cranioplasty were significantly lower after RA as compared with RQM technique; however, in light of the consistent failure to reproduce this finding in the other studies, or for the pooled effect size to reach statistical significance, this more likely represents a spurious finding than true signal. More specifically, Hoffman et al. and Früh et al. observed essentially comparable infection rates after RQM versus RA procedures, while Dowlati et al. noted a lower infection rate after RA, 8.3% as compared with 14%, although that difference was not statistically significant. 3 6 9

In addition to SSI, impactful considerations for DHC operations include the extent of decompression, non-infectious wound complications such as poor healing or bone flap resorption, and long-term recovery after DHC. Although some authors noted differences in these outcomes between the RQM and RA approaches, such as the larger decompression sizes after RA incision (average 9.2 cm ^2^ larger) by Dowlati et al., the pooled estimates were not significantly different across the meta-analysis. 3 6 9 Further, on any such variable, no more than one study reported a significant difference that diverged from the non-significant findings in all the other studies, for example, with Früh et al. and Hoffman et al., both finding no benefit in the extent of decompression between the techniques.

Interestingly, the only significant difference between RQM and RA patients noted in the pooled estimates derived from the present study was that a higher proportion of RQM patients went on to subsequent cranioplasty. Given the lack of a companion association with a wound complication, our inference is that this likely reflects a difference in underlying injury severity that cannot be assessed based on the relatively limited patient-level data provided by the included studies.

### Considerations for Surgical Planning

Perhaps the most important conclusion to be drawn from the lack of a significant difference across wound outcomes between RQM and RA techniques in our pooled analysis is the utility of both approaches across a range of clinical contexts. Put differently, the current study supports either technique as a safe default strategy based on surgeon preference; however, the incorporation of the theoretical strengths and limitations of each may inform decision-making in atypical patient-specific contexts.

Defenders of the RA technique emphasize the importance of STA preservation, which in turn supplies a robust, multivessel blood supply to the flap. Although a well-executed RQM should still preserve at least the frontal branch of the STA, the parietal branch is frequently sacrificed, and so this inference is at least bioplausible—if not conclusively supported by the reported data. Notwithstanding, one can imagine that the benefits of complete STA preservation might be augmented in a patient who is at specially elevated risk of a wound complication, such as a brittle diabetic, a patient with metastatic malignancy, or a patient with poor scalp tissue due to an unrelated disease process such as psoriasis or severe alopecia. Under such conditions, the RA technique would be reasonably favored, especially in light of the non-inferior findings in the present study.

By token, the major concern regarding the RA incision is difficulty with accessing the anterior–inferior aspect of the supratentorial intracranial compartment. For optimal decompression, the squamosal temporal bone is removed down to the level of the middle fossa floor, which relieves pressure on the midbrain by allowing for optimal decompression of the uncus. Although the reported results appear to indicate equivalence between the extent of decompression, anecdotal evidence and the results reported by Dowlati et al. indicate that this is a potential pitfall of the approach. 3 Correspondingly, in a decompression where the patient has blown a pupil and rapid, complete relief of uncal herniation is mandatory, we would strongly favor the RQM technique. Less concerningly, RA incisions may bear an increased risk of rare complications such as violation of the external auditory canal, CSF leak via exposed mastoid air cells, or damage to the transverse or sigmoid sinus if the posterior aspect of the craniotomy is extended too far inferior. 11 Although these complications have been reported, they most likely reflect unusual circumstances or lack of familiarity with the technique and associated anatomy, which would be readily addressed by more comprehensive training on both techniques among neurosurgical residents.

## Future Research

The current study represents the second recent meta-analysis exploring the relationship between the RQM versus RA, which through complementary study designed reached mutually validating conclusions about the optimal clinical role of these techniques. In their recent work, Brown et al. 12 also incorporated the Kempe incision but focused exclusively on primary SSI and other early wound complications without attention to secondary outcomes after cranioplasty. They found no significant difference between the RQM and RA techniques for primary DHC, 12 despite the theoretical advantages afforded by the putatively superior blood supply to the flap. With these considerations in mind, future studies along this same avenue of inquiry might explore subgroups for whom RQM or RA might be especially beneficial, as detailed above, to support or reject our hypotheses with meaningful clinical data. Still, further studies could specifically assess pediatric patients, high-risk adults, and less common indications such as herniation from a tumor of a spontaneous hemorrhage that requires urgent surgical decompression.

## Strengths and Limitations

The current study is subject to the full swath of systematic errors that apply broadly to both observational data and meta-research. Further, the analysis is based on a small sample of studies, each of which captures a modest number of patients, resulting in several opportunities for sampling bias. Although most of the included studies did not deliberately recruit narrow or unusual patient samples; however, the lack of patient-level data makes rendering nuanced conclusions in that regard impossible, and the generalizability of our results to other important populations such as children or elderly individuals with high-risk medical conditions is also limited. None of the included manuscripts incorporated randomization, and so the choice to proceed with RQM- versus RA-based DHC is entirely based on surgeon preferences and, therefore, represents several additional opportunities for selection, sampling, or referral bias—among other potential sources of error.

Despite these compromises, our work represents a unique and timely contribution to the literature on an interesting and clinically impactful question. Ours is the first such meta-analysis to include data from all pertinent phases of care and strictly limit inclusion to those studies reporting primary head-to-head comparisons between the surgical techniques. This is particularly useful in establishing how two surgical techniques compare with each other, allowing for better clinical decision-making. These methodological choices lend robustness to our results, and when taken together with the congruent findings from preceding analyses, the study results provide strong and compelling data in support of either RQM or RA as a safe, effective, and low-risk technique for adult patients undergoing DHC.

## Conclusion

We report a novel systematic review and meta-analysis comparing RQM and RQ incisions for DHC in the adult population. Neither technique was associated with a significant increase in risk of primary SSI, secondary SSI, or other wound complication. Overall, these results support the contemporary practice of deferring to surgeon preference and expertise in adjudicating a decision on the final treatment plan; however, for certain populations at especially increased risk of SSI, uncal herniation, or other highly salient clinical conditions, RQM- or RA-based strategies may warrant preferential consideration on a case-by-case basis.
